# Supplementary material for: Risk factors for hydrocephalus following fourth ventricle tumor surgery: A retrospective analysis of 121 patients
Source: PLoS One. 2020 Nov 17;15(11):e0241853. doi: 10.1371/journal.pone.0241853 (PMC7671531; doi:10.1371/journal.pone.0241853)
Supplement: S1 Table — (PDF) [file pone.0241853.s001.pdf]

**Variables**	**Value**
Sex	
Female	61 (50.4%)
Male	60 (49.6%)
Tumor size (mm)	37 (30-44)
Age (years)	24 (9-41)
<3	11 (9.1%)
3–5	10 (8.3%)
5–18	35 (28.9%)
>18	65 (53.7%)
Tumor pathology	
Ependymoma	37 (30.6%)
Medulloblastoma	29 (24.0%)
Astrocytoma	20 (16.5%)
Hemangioblastoma	7 (5.8%)
Cholesteatoma	7 (5.8%)
Choroid plexus papilloma	7 (5.8%)
Metastatic	4 (3.3%)
Non-Hodgkin lymphoma	1 (0.8%)
Other	9 (7.5%)
Tumor growth characteristics [a]	
Lateral extension	34 (28.1%)
Anterior extension	98 (81.0%)
Caudal extension	69 (57.0%)
Superior extension	14 (11.6%)
No extension beyond the fourth ventricle	8 (6.6%)

Values are number of patients (%) or median (interquartile range).

[a] Percentages do not add up to 100 because some patients had more than 1 growth characteristic.
