# Supplementary material for: Risk factors for hydrocephalus following fourth ventricle tumor surgery: A retrospective analysis of 121 patients
Source: PLoS One. 2020 Nov 17;15(11):e0241853. doi: 10.1371/journal.pone.0241853 (PMC7671531; doi:10.1371/journal.pone.0241853)
Supplement: S2 Table — (PDF) [file pone.0241853.s002.pdf]

**Variables**	**Postoperative CSF diversion**		**P-value**
	Yes (15)	No (106)	
Sex			0.012[a]
Female	3 (4.9%)	51 (95.1%)	
Male	12 (20%)	48 (80%)	
Tumor size (mm)	40 (34-49)	36 (30-43)	0.100
Age (years)	18 (3-38)	24 (9-41)	0.301
Tumor pathology			
Ependymoma	3 (8.1%)	34 (91.9%)	0.226[b c]
Medulloblastoma	5 (17.2%)	24 (82.8%)	1.0[b c]
Astrocytoma	4 (20.0%)	16 (80.0%)	
Lateral extension			0.553[b]
Yes	3 (8.8%)	31 (91.2%)	
No	12 (13.8%)	75 (86.2%)	
Anterior extension			0.159[b]
Yes	88 (89.8%)	10 (10.2%)	
No	5 (21.7%)	18 (78.3%)	
Caudal extension			0.420[a]
Yes	10 (14.5%)	59 (85.5%)	
No	5 (9.6%)	47 (90.4%)	
Superior extension			0.015[b]
Yes	5 (35.7%)	9 (64.3%)	
No	10 (9.3%)	97 (90.7%)	
Extent of resection			<0.001[b]
GTR	3 (3.3%)	87(96.7%)	
STR	12 (38.7%)	19(61.3%)	
Preoperative hydrocephalus			<0.001[a]
Yes	15 (21.4%)	55 (78.6%)	
No	0 (0%)	51 (100%)	
Prophylactic EVD			0.091[a]
Yes	10 (17.9%)	46 (82.1%)	
No	5 (7.7%)	60 (92.3%)	

CSF, cerebrospinal fluid; GTR, gross total resection; STR, subtotal resection; EVD, external ventricular drainage.

[a] Chi-square test.

[b] Fisher exact test.

[c] p value compared with astrocytoma
