# Supplementary material for: Risk factors for hydrocephalus following fourth ventricle tumor surgery: A retrospective analysis of 121 patients
Source: PLoS One. 2020 Nov 17;15(11):e0241853. doi: 10.1371/journal.pone.0241853 (PMC7671531; doi:10.1371/journal.pone.0241853)
Supplement: S3 Table — (PDF) [file pone.0241853.s003.pdf]

**Variables**	**Postoperative VP**		**P-value**
	Yes (10)	No (110)	
Sex			0.054[b]
Female	2 (3.3%)	59 (96.7%)	
Male	8 (13.3%)	52 (86.7%)	
Tumor size (mm)	44 (33-53)	36 (30-43)	0.032
Age (years)	16 (1-45)	24 (9-41)	0.533
Tumor pathology			
Ependymoma	2 (5.4%)	35 (94.6%)	0.170[b c]
Medulloblastoma	3 (10.3%)	26 (89.7%)	0.422[b c]
Astrocytoma	4 (20.0%)	16 (80.0%)	
Lateral extension			0.724[b]
Yes	2 (5.9%)	32 (94.1%)	
No	8 (9.2%)	79 (90.8%)	
Anterior extension			0.339[b]
Yes	7 (7.1%)	91 (92.9%)	
No	3 (13%)	20 (87%)	
Caudal extension			1.000[b]
Yes	6 (8.7%)	63 (91.3%)	
No	4 (7.7%)	48 (92.3%)	
Superior extension			0.016[b]
Yes	4 (28.6%)	10 (71.4%)	
No	6 (5.6%)	101 (94.4%)	
Extent of resection			<0.001[b]
GTR	1 (1.1%)	89(98.9%)	
STR	9 (29%)	22(71%)	
Preoperative hydrocephalus			<0.005[b]
Yes	10 (14.3%)	60 (85.7%)	
No	0 (0%)	51 (100%)	
Prophylactic EVD			0.006[b]
Yes	9 (16.1%)	47 (83.9%)	
No	1 (1.5%)	64 (98.5%)	

[a] Chi-square test.

[b] Fisher exact test.

[c] p value compared with astrocytoma
