# Supplementary material for: Risk factors for hydrocephalus following fourth ventricle tumor surgery: A retrospective analysis of 121 patients
Source: PLoS One. 2020 Nov 17;15(11):e0241853. doi: 10.1371/journal.pone.0241853 (PMC7671531; doi:10.1371/journal.pone.0241853)
Supplement: S4 Table — (PDF) [file pone.0241853.s004.pdf]

**Variables**	**Odds ratio (95% CI)**	**p-value**
Superior extension	44.761(2.235-896.310)	0.013
Gross total resection	0.087(0.016-0.473)	0.005
