# Supplementary material for: Risk factors for hydrocephalus following fourth ventricle tumor surgery: A retrospective analysis of 121 patients
Source: PLoS One. 2020 Nov 17;15(11):e0241853. doi: 10.1371/journal.pone.0241853 (PMC7671531; doi:10.1371/journal.pone.0241853)
Supplement: S5 Table — (PDF) [file pone.0241853.s005.pdf]

**Variables**	**Postoperative CSF diversion**		**p-value**
	Yes (10)	No (46)	
Sex			0.162[b]
Female	2(8.3%)	22(91.7%)	
Male	8(25.0%)	24(75.0%)	
Tumor size (mm)	41(33-50)	36(31-41)	0.174
Age (years)	22.5(1-45)	25.5(11-41)	0.528
Tumor pathology			
Ependymoma	3(20.0%)	12(80.0%)	0.239[b c]
Medulloblastoma	2(15.4%)	11(84.6%)	0.357[b c]
Astrocytoma	4(36.4%)	7(63.6%)	
Lateral extension			0.713[b]
Yes	2(13.3%)	13(86.7%)	
No	8(19.5%)	33(80.5%)	
Anterior extension			0.361[b]
Yes	7(15.2%)	39(84.8%)	
No	3(30.0%)	7(70.0%)	
Caudal extension			0.727[b]
Yes	7(20.0%)	28(80.0%)	
No	3(14.3%)	18(85.7%)	
Superior extension			0.003[b]
Yes	5(62.5%)	3(37.5%)	
No	5(10.4%)	43(89.6%)	
Extent of resection			<0.001[b]
GTR	2(5.0%)	38(95.0%)	
STR	8(50.0%)	8(50.0%)	

[a] Chi-square test.

[b] Fisher exact test.

[c] p value compared with astrocytoma
