# Supplementary material for: Risk factors for hydrocephalus following fourth ventricle tumor surgery: A retrospective analysis of 121 patients
Source: PLoS One. 2020 Nov 17;15(11):e0241853. doi: 10.1371/journal.pone.0241853 (PMC7671531; doi:10.1371/journal.pone.0241853)
Supplement: Attachment — Submitted filename: Response to Reviewers.docx [file pone.0241853.s008.docx]

Dear Editors:

Thank you for your letter and comments concerning our manuscript entitled ***"*****Risk factors for hydrocephalus following fourth ventricle tumor surgery: A retrospective analysis of 121 patients***"* (PONE-D-20-23644). We have studied the comments carefully and made corresponding modifications. All modifications in the manuscript were highlighted in red. The main corrections in the paper and the responses to the reviewers’ and editor’s comments are as follows:

Reviewers’ comments:

Reviewer #1

1. This study is a retrospective chart review that addresses the incidence of persistent postoperative hydrocephalus requiring surgery, in patients undergoing surgery for resection of fourth ventricular tumors. The study adds significantly to existing knowledge about this issue. The study includes a large number of patients, who were treated using a relatively standardized protocol. The analysis was thoughtfully designed and uses statistics appropriately. I believe that this study will be useful for practicing neurosurgeons, in order to counsel patients and their families appropriately about the likelihood of needing additional surgeries (ETV or shunt) to treat hydrocephalus, after resection of 4th ventricular tumors, and in order to assess the likelihood of EVD weaning after these surgeries.

**Authors’ response: Thank you for the comments.**

Reviewer #2

1. The manuscript is well written and easy to read.

**Authors’ response: Thank you for the comment.**

2. The topic is relevant and of general interest.

**Authors’ response: Thank you for the comment.**

3. My main objection to this manuscript is the choice of main outcome variable. In my opinion, the combination of post-resection EVD and permanent VP is confusing, all the time 56/121 had placement of EVD before resection. I would have preferred to use permanent VP alone as main outcome variable. Doing this, pre-operative HC will most likely remain significantly associated with post-resection VP placement. I encourage the authors to at least include such an analysis. It is of course interesting to note that 5/65 patients without pre-resection placement of EVD needed EVD in the early postoperative period, but only one of these five ended up with VP.

**Authors’ response: We have added this analysis. (Page8, line172-179)**

4. Identifying superior extension and subtotal resection as independent risk factors for post resection CSF drainage (EVD or VP-shunt) is absolutely of clinical interest, but will be even more interesting if analyzed according to the comment above.

**Authors’ response: It has been analyzed according to the comment above.**

5. Pre-resection endoscopic third ventriculostomy (ETV) is an alternative to EVD, and may reduce the need for permanent VP. The mention of ETV in the discussion is somewhat brief.

**Authors’ response: We have added some details about EVT in the discussion. (Page13, line 264-274)**

Reviewer #3

1. The authors have sufficiently addressed my concerns and feel that with the changes the article is not suitable for publication. The authors have significantly enhanced the discussion section that was previously lacking.

**Authors’ response: Thank you for your comment. But we did not make a revision before.**

Reviewer #4

1. This is a nice series which outlines the 4th ventricular tumor situations which are more likely to lead to persistent hydrocephalus requiring shunting. The authors are to be congratulated on this analysis.

**Authors’ response: Thank you for the comment.**

Reviewer #5

1. what were indications for preoperative EVD placement and timing for such procedure in relation to tumor surgery

**Authors’ response: We have described the indications in the sentence (Page5, line 114-116). And we have added details about the timing for preoperative EVD placement (Page5, line 116-118).**

2. What was the timing for definitive CSF diversion surgery in relation to the index tumor operation

**Authors’ response: We have described them in the sentence (Page 5, line 126-128).**

3. What was incidence of complications with the shunt procedure

**Authors’ response: No serious complications occurred in any of the cases of VP shunting during the follow-up period.**

4. What was the shunt infection rate?

**Authors’ response: No patient with shunt infection was found during the follow-up period.**

5. Did the authors consider an ETV rather than VP shunt

**Authors’ response: Thank you for your valuable comment. We did not routinely perform ETV when the patients in this study were treated for hydrocephalus. But now, ETV was performed more than VP in our hospital. Moreover, the type of surgery for postoperative hydrocephalus do not affect the occurrence of postoperative hydrocephalus as well as the risk factors.**

Reviewer #6

1. The median age of the patient population is 24 years of age and this should be more explicitly mentioned, perhaps even in the title of the manuscript, as typically fourth ventricular tumors tend to be more highly concentrated in a pediatric population.

**Authors’ response: We have explicitly mentioned and added details about age in the outcome. (Table 1)**

2. The conclusions from the paper state that superior tumor extension and failed gross total resection were independent risk factors for VPS placement. However, this makes one wonder whether a subtotal resection within the aqueduct led to hydrocephalus. If this is the case then this is an obvious conclusion in that the obstruction through the ventricular system was not alleviated. However, if indeed, superior extension and failed total resection were not factors related to the same patients then this needs to be further explained in the manuscript and the manuscript would benefit from the authors’ hypotheses on how this would then lead to hydrocephalus. In addition to having a data analysis on initial extension of the tumor on patient presentation, a further explanation of which areas of tumor were left behind when a gross total resection was not possible would also assist in explaining the results.

**Authors’ response: I am sorry we did not mention this information. There were no cases with the residual tumor in the aqueduct. During the operation, the surgeons ensured regarding the adequacy of visualization of the aqueduct and CSF flow after tumor removal. Meanwhile, there is no residual tumor near the aqueduct and the residual tumors are located in the brain stem and the bottom of the fourth ventricle in failed-total resection cases on postoperative MRI. We have added this sentence (Page 14, line 296-299).**

3. Further, the authors mentioned that endoscopic third ventriculostomy (ETV) was a consideration for patients with fourth ventricular obstruction. The paper does not address why none of the patients who eventually underwent VPS placement were or were not considered for ETV as this may have been able to avoid permanent CSF diversion through a shunt.

**Authors’ response: We do not routinely use ETV for hydrocephalus with fourth ventricle tumor, and this will be improved in the future. Thank you for this valuable comment.**

Reviewer #7

1. There is a basic assumption in this manuscript which may be fallacious: Regardless of pathology it is the anatomic extent of the tumor that determines hydrocephalus. Although using appropriate statistical analysis the office did not demonstrate significance of pathology, the subgroup's may be too small to provide a true "real world answer".

**Authors’ response: Thank you for this valuable comment. We have mentioned this in the limitations. (Page, line 282-294)**

2. In the discussion the role of age and its implication for development of hydrocephalus is briefly reviewed but I believe this needs to be further expanded. It is not just age but the more aggressive nature of the tumors that we see in children under 5 years of age and particularly under 3 years of age with leptomeningeal dissemination that may increase the risk of hydrocephalus. The older patients in this study and the relatively small number of those with medulloblastoma may give a false impression of watch factors truly influence the development of hydrocephalus.

**Authors’ response: We have further expanded the part in the discussion (Page 13, line 230-240). Thank you for this valuable comment.**

3. I would be curious if this finding would hold up if the authors limited the population studied 2 patients over 16 years of age, that is those that are anatomically adult. I am not certain that this finding would be seen in younger children, particular with medulloblastoma where the mean age is below that of the study population in this manuscript.

**Authors response: Superior tumor extension and subtotal resection were identified as independent risk factors for hydrocephalus after resection of tumor in the fourth ventricle in this study. But only subtotal resection was significantly associated with hydrocephalus when only patients older than 16 years were enrolled. This seems to be a type II error, which might be attributed to a smaller sample size when pediatric patients were excluded.**

Finally, we earnestly appreciate the Editor’s helpful work and hope that these answers and corrections will meet with approval. Once again, thank you very much for your comments and suggestions.

Best regards.

Yuekang Zhang

Department of Neurosurgery, West China Hospital, Sichuan University, China

E-mail: 2012zykyx@sina.cn
