# Supplementary material for: Risk factors for hydrocephalus following fourth ventricle tumor surgery: A retrospective analysis of 121 patients
Source: PLoS One. 2020 Nov 17;15(11):e0241853. doi: 10.1371/journal.pone.0241853 (PMC7671531; doi:10.1371/journal.pone.0241853)
Supplement: Attachment — Submitted filename: Response to Reviewers.docx [file pone.0241853.s009.docx]

Dear Editors:

Thank you for your letter and comments concerning our manuscript entitled ***"*****Risk factors for hydrocephalus following fourth ventricle tumor surgery: A retrospective analysis of 121 patients***"* (PONE-D-20-23644R1). We have studied the comments carefully and made corresponding modifications. All modifications in the manuscript were highlighted in red. The main corrections in the paper and the responses to the reviewers’ and editor’s comments are as follows:

Editor’s comments：

1. Please add a paragraph into the discussion part of the manuscript where you explain whether or not less than 1.5 cc of residual tumor coating the floor of the fourth ventricle will necessitate ultimate CSF diversion.

**Authors’ response: We have further expanded the part in the discussion (Page 14, line 288-298). Thank you for this valuable comment.**

Reviewers’ comments:

Reviewer #1

1. I recommend publication in its current form

**Authors’ response: Thank you for the comments.**

Reviewer #2

1. In my opinion, the authors have responded well to the reviewers comments, and the revised manuscript is improved compared to the original manuscript.

**Authors’ response: Thank you for the comment.**

Reviewer #4

1. I believe the revisions improve the quality of the article. I believe the authors have answered the reviewers concerns to my satisfaction.

**Authors’ response: Thank you for the comment.**

Reviewer #5

(No Response)

Reviewer #7

1. I still take issue with the comment that a gross total resection versus a near total resection is a significant indicator for the need of a shunt procedure particularly in younger children. I would urge the authors to further expand the discussion, particularly questioning whether less than 1.5 cc of residual tumor coating the floor of the fourth ventricle will necessitate ultimate CSF diversion. This is particularly pertinent since it has been well-established in the pediatric neuro-oncology literature that there is no survival advantage for gross total resection versus minimal residual disease.

**Authors’ response: We have further expanded the part in the discussion (Page 14, line 288-298). Thank you for this valuable comment.**

Finally, we earnestly appreciate the Editor’s helpful work and hope that these answers and corrections will meet with approval. Once again, thank you very much for your comments and suggestions.

Best regards.

Yuekang Zhang

Department of Neurosurgery, West China Hospital, Sichuan University, China

E-mail: 2012zykyx@sina.cn
